# The Hsp90-Dependent Proteome Is Conserved and Enriched for Hub Proteins with High Levels of Protein–Protein Connectivity

**DOI:** 10.1093/gbe/evu226

**Published:** 2014-10-13

**Authors:** Rajaneesh Karimpurath Gopinath, Shu-Ting You, Kun-Yi Chien, Krishna B.S. Swamy, Jau-Song Yu, Scott C. Schuyler, Jun-Yi Leu

**Affiliations:** ^1^Molecular and Cell Biology, Taiwan International Graduate Program, Graduate Institute of Life Sciences, National Defense Medical Center and Academia Sinica; ^2^Institute of Molecular Biology, Academia Sinica, Taipei, Taiwan; ^3^Molecular Medicine Research Center, Department of Biochemistry and Molecular Biology, College of Medicine, Chang Gung University, Tao-Yuan, Taiwan; ^4^Department of Cell and Molecular Biology, College of Medicine, Chang Gung University, Tao-Yuan, Taiwan; ^5^Department of Biomedical Sciences, College of Medicine, Chang Gung University, Tao-Yuan, Taiwan

**Keywords:** molecular chaperone, genetic buffering, yeast proteomics, protein network, yeast genomics

## Abstract

Hsp90 is one of the most abundant and conserved proteins in the cell. Reduced levels or activity of Hsp90 causes defects in many cellular processes and also reveals genetic and nongenetic variation within a population. Despite information about Hsp90 protein–protein interactions, a global view of the Hsp90-regulated proteome in yeast is unavailable. To investigate the degree of dependency of individual yeast proteins on Hsp90, we used the “stable isotope labeling by amino acids in cell culture” method coupled with mass spectrometry to quantify around 4,000 proteins in low-Hsp90 cells. We observed that 904 proteins changed in their abundance by more than 1.5-fold. When compared with the transcriptome of the same population of cells, two-thirds of the misregulated proteins were observed to be affected posttranscriptionally, of which the majority were downregulated. Further analyses indicated that the downregulated proteins are highly conserved and assume central roles in cellular networks with a high number of protein interacting partners, suggesting that Hsp90 buffers genetic and nongenetic variation through regulating protein network hubs. The downregulated proteins were enriched for essential proteins previously not known to be Hsp90-dependent. Finally, we observed that downregulation of transcription factors and mating pathway components by attenuating Hsp90 function led to decreased target gene expression and pheromone response, respectively, providing a direct link between observed proteome regulation and cellular phenotypes.

## Introduction

Proteins in the cell have to fold into their active conformation to perform defined functions. Protein folding is a fundamental task within a cell. Maintenance of protein homeostasis (or “proteostasis”) is challenging, due to the “molecular crowding” environment created in the cell by the plethora of macromolecules (Ellis 2001). Cells may encounter proteotoxic stresses, such as heat and chemicals, which lead to protein misfolding. Accumulation of toxic aggregates formed by misfolded proteins has been speculated to cause many neurodegenerative diseases, such as Alzheimer’s and Parkinson’s, illustrating the critical function of protein folding and stability in cell physiology (Dobson 2001; Ross and Poirier 2004). Sustaining the balance between protein synthesis, maintenance, and degradation is a demanding task and it often requires direct specialized help from molecular chaperones, which aid in the efficient folding of nascent polypeptides and refolding of proteins upon activation (Hartl 1996; Hartl and Hayer-Hartl 2002). Eukaryotes have developed two separate molecular chaperone systems: One for protein biogenesis and the other that is stress-induced to prevent protein aggregation (Albanese et al. 2006). Molecular chaperones also partner with the ubiquitin–proteasome system to degrade irreparably aggregated proteins and function in intracellular protein transport (Young et al. 2003; Qbadou et al. 2006).

Heat shock proteins (HSPs) constitute the major family of the molecular chaperones that require ATP hydrolysis for protein folding (Frydman 2001; Young et al. 2004; Saibil 2013). Among them, Hsp90 is of particular interest because it is one of the most abundant proteins in the cell and is highly conserved from bacteria to higher eukaryotes (Chang and Lindquist 1994). Hsp90 is a homodimeric ATPase whose major function is to assist in the late stage folding of metastable proteins (Nathan et al. 1997). With the aid of cochaperones, Hsp90 binds to a variety of clients and is involved in various biological processes (Taipale et al. 2010). Hsp90 family proteins contain three main domains, a N-terminal domain required for ATP binding, a Middle (M) domain, and a C-terminal domain for dimerization. The catalytic groups of both the N-terminal and M domains contribute to ATP hydrolysis, making Hsp90 a “split ATPase” (Meyer et al. 2003). Inhibitory drugs, such as geldanamycin and radicicol, bind to the ATP-binding pocket of the N-terminal domain, which prevents ATP hydrolysis and inactivates Hsp90 function (Roe et al. 1999). The C-terminal domain consists of the conserved MEEVD pentapeptide motif with which it binds to many cochaperones harboring the tetratricopeptide repeat (TPR) domains (Chen et al. 1998; Young et al. 1998). There are exceptions to this mechanism of interaction, like the Sgt1 cochaperone that does not bind to Hsp90 through the TPR domain (Lee et al. 2004). Due to the absence of a general interaction pattern and the existence of numerous target-specific cochaperones, the requirements for the formation of a particular substrate–cochaperone–Hsp90 complex are still unclear (Caplan et al. 2007; Eckert et al. 2010; Zhang et al. 2010).

In addition to maintaining protein homeostasis, Hsp90 has also been suggested to buffer genetic variations, thereby linking chaperones in shaping the evolutionary processes (Rutherford and Lindquist 1998; Queitsch et al. 2002; Cowen and Lindquist 2005). Because mutations in the coding region often cause inappropriate protein folding and compromise gene function, the energy-dependent help from chaperones in protein folding may allow cells to completely or partially restore the mutant protein function and therefore mask the effects of mutations (described as the “direct buffering” hypothesis) (Fares et al. 2002; Sangster et al. 2004; Lauring et al. 2013). The “cryptic” mutations are expected to accumulate in a population under normal conditions, but reveal their effects to assist population adaptation when Hsp90 is depleted (Sangster et al. 2004). In some cases, Hsp90 buffering facilitates the accumulation of variations to the extent where existing traits are improved or new ones evolve (Cowen and Lindquist 2005; Rohner et al. 2013). Because many Hsp90 clients are tumor-related proteins and cancer development is a process of cellular evolution, the effect of Hsp90 buffering is also suggested to impact tumorigenesis (Whitesell and Lindquist 2005; Miyata et al. 2013). However, the underlying genetic bases of Hsp90 buffering are mostly unexplored (Jarosz and Lindquist 2010). Though the effects and implications of Hsp90-mediated capacitance have been observed in several species, the evolutionary impact of Hsp90 buffering remains elusive (Jarosz et al. 2010).

In our recent study, Hsp90 was demonstrated to regulate nongenetic variation (stochastic variation) in yeast cells (Hsieh et al. 2013). In wild-type cells, Hsp90 insulates against the impact of stochastic fluctuations by maintaining the abundance of two morphogenesis regulators in a constant and above-threshold level. However, when the activity or expression of Hsp90 is compromised, these regulators are reduced to near threshold levels, resulting in morphological heterogeneity in the population. Previous studies on pathogen treatments or cancer therapies have suggested that population heterogeneity caused by nongenetic variation may play an important role for the survival of pathogens or cancer cells during treatment (Lewis 2007; Brock et al. 2009). It will thus be interesting to determine how many pathways Hsp90 influences through nongenetic variation, especially under stressful conditions.

Previous large-scale profiling studies in yeast have established that the Hsp90 interactome covers 10–20% of the yeast proteome (Millson et al. 2005; Zhao et al. 2005; McClellan et al. 2007; Gong et al. 2009; Franzosa et al. 2011). These studies comprehensively investigated the physical, genetic, and chemical-genetic interactome of Hsp90 and found that the putative interactors of Hsp90 are enriched in the pathways of transcriptional regulation, cell cycle, DNA processing, and cellular transport. However, the results of these studies are disparate with each other (Hartson and Matts 2012). Besides, some known physical interactors are missing in these studies, probably because their interactions are transient or are too weak to be detected (Hsieh et al. 2013). Most importantly, despite this deluge of information on Hsp90 interactors, in many cases, we still cannot pinpoint the underlying mechanism of the observed cellular phenotypes caused by the reduced Hsp90 activity. The extent to which an interactor depends on Hsp90 is variable, thus it is likely that under a certain condition only a subset of the interactors are influenced to the degree that leads to phenotypic changes. To fill the gap between the interactome data and observed cellular phenotypes in low-Hsp90 cells, a comprehensive study uncovering the Hsp90-dependent proteome, with a clear distinction from transcriptional regulation, is warranted.

Combining stable isotope labeling by amino acids in cell culture (SILAC) with mass spectrometry (MS) is a powerful strategy for investigating the global influence of Hsp90 on the proteome (Mann et al. 2013). Recent studies have applied this strategy to provide quantitative analyses of Hsp90-dependent proteome in human cell lines (Sharma et al. 2012; Wu et al. 2012; Fierro-Monti et al. 2013). Nonetheless, the ease of genetic manipulation, availability of well-annotated genomic data, and the capacity to attain better protein coverage make yeast an advantageous system for a detailed characterization in whole proteome studies (de Godoy et al. 2006). Moreover, Hsp90 is highly conserved with 60% identity between yeast and humans and a majority of Hsp90’s interactors in yeast have human orthologs. Thus, the information learned from yeast may not only allow us to derive the general features of Hsp90-dependent proteome but also complement the knowledge obtained from human studies.

In this study, our goal was to measure the extent of dependency of the yeast proteome on Hsp90 and to examine the general features of Hsp90-dependent proteins. We used SILAC-coupled MS to quantify changes in cellular protein abundance in low-Hsp90 yeast cells. We concurrently performed a genome-wide analysis of messenger RNA (mRNA) isolated from the same population of low-Hsp90 cells to assess the proportion of yeast genes affected by Hsp90 transcriptionally. Our results revealed that about 11% of detected proteins have reduced abundance significantly without a change in their mRNA levels. The downregulated proteins of low-Hsp90 cells are highly conserved and assume central roles in complex protein–protein interaction networks. Higher sequence conservation was also observed in the downregulated proteins of human low-Hsp90 cells, suggesting that this feature is general across species. Finally, we experimentally validated that downregulated proteins could lead to changes in cellular behaviors when the Hsp90 activity was compromised.

## Materials and Methods

### Strains, Genetic Procedures, and Growth Conditions

The endogenous promoter of *HSC82* was replaced by the *tet*O7 promoter (from the pUC*tet*O7 plasmid) in the S288C strain background. This strain was crossed with an *hsp82Δ* mutant in the same background. The resulting diploid cells were sporulated to generate the haploids with the correct genotype (*hsp82Δ TETp-HSC82 ura3::tTA*). The arginine and lysine double auxotroph of the *TETp-HSC82* strain was constructed by deleting the *ARG4* and *LYS5* genes, respectively. In addition, the *CAR2* gene was deleted to resolve the arginine conversion problem typically encountered in SILAC studies (Bicho et al. 2010). The green fluorescent protein (GFP)-tagged genes were obtained from the yeast GFP collection (Huh et al. 2003).

For SILAC labeling, the *TETp-HSC82 hsp82Δ arg4Δ lys5Δ car2Δ::URA3* cells (the SILAC strain) were grown in 2-fold modified complete synthetic medium (2× CSM) which contains double the amount of amino acid supplements containing either 40 mg/l l-arginine and 60 mg/l l-lysine (light labeling) or 40 mg/l ^13^C_6_ l-arginine and 60 mg/l ^13^C_6_ l-lysine (heavy labeling). We then treated one population of cells with 5 μg/ml doxycycline (dox) (Sigma-Aldrich, St. Louis, MO) dissolved in sterilized ddH_2_O, for 11 h. Cells were kept in log phase for the whole process. During this period, the growth difference between control and dox-treated populations was less than one cell division. The light- and heavy-labeled cells were treated with dox in the first and second SILAC experiments, respectively.

For FACS validation of protein abundance, GFP strains were grown to log phase in 2× CSM. We then treated cells with 50 μM macbecin II (National Cancer Institute/National Institute of Health, USA) for 4 h and then analyzed GFP signals using a BD FACSJazz machine (Becton-Dickinson Biosciences, Franklin Lake, NJ).

### Lysis and Protein Extract Preparation

Normal and heavy SILAC-labeled yeast cells were mixed in a 1:1 ratio of cell numbers. The cells were resuspended in the lysis buffer (25 mM HEPES pH 7.5, 10 mM NaCl, 1 mM phenylmethanesulfonyl fluoride, and 1 mM dithiothreitol [DTT]) and lysed using 0.5-mm glass beads with five beating cycles of 30-s working and 30-s cooling. To the mixture, 0.1% sodium dodecyl sulfate (SDS) was added and mixed by vortexing. All the above steps were performed at 4 °C. The cell lysate was ultracentrifuged to remove the undissolved materials, and the protein was quantified using bicinchoninic acid assay (BCA1; Sigma-Aldrich). We aliquoted protein samples into 50 μg per each tube, and stored them at −80 °C before MS analyses.

### Trypsin Digestion and Sample Clean-Up

Samples containing 50 μg protein were dissolved in 20 μl of 50 mM NH_4_HCO_3_ containing 0.1% SDS. Each sample was reduced by 5 mM DTT at 56 °C for 30 min and then alkylated by 15 mM iodoacetamide at room temperature for 30 min. Excess iodoacetamide was quenched by additional 5 mM DTT at room temperature for 30 min. The solution was then brought to 200 μl with 10 mM NH_4_HCO_3_, and digestion was performed in the presence of 0.5 μg trypsin (Promega, Madison, WI) at 37 °C overnight. The resulting peptide mixture was cleaned-up by a SOURCE 15RPC reverse phase (RP) microcolumn (GE Healthcare, Uppsala, Sweden) and dried in a SpeedVac concentrator.

### Online Two-Dimensional Liquid Chromatography

The comprehensive two-dimensional (2D)-strong cation exchange (SCX)-RP-liquid chromatography (LC) system (Ultimate 3000; Thermo Fisher Scientific/Dionex, Germering, Germany) has been equipped with two gradient pumps (one for first- and the other for second-dimensional separation), one isocratic pump, one 10-port valve (installed with two RP-trapping columns), and one 6-port valve. The online 2D peptide separation was achieved by operating both gradient pumps simultaneously, and switching the 10-port valve every 65 min during the entire analysis. Briefly, protein digests dissolved in 50% acetonitrile containing 0.1% formic acid were loaded onto the SCX column (0.5 × 150 mm, pack with Luna-SCX particles from Phenomenex, Torrance, CA), which was operated at a flow rate of 1.5 μl/min. Peptides were eluted using a continuous concentration gradient of ammonium chloride in the presence of 0.1% formic acid and 30% acetonitrile. The salt gradient was segmented in 44 steps, 65 min for each, and eluted peptides from each step were then separated by the second-dimensional RP column (0.075 × 150 mm, pack with Synergi Hydro-RP particles from Phenomenex). The isocratic pump delivering 70 μl/min of solvent A (0.1% formic acid in water) was used for diluting the effluent of SCX column through a T-union and mixing tubing before it reached the trapping column (0.5 × 5 mm, packed with Symmetry300 C18 particles from Waters, Milford, MA). In the meantime, the other RP-trapping column was connected to the RP-analytical column and the effluent was analyzed by a mass spectrometer. Five minutes before each salt gradient step being completed, the gradient pump for the SCX separation stopped, and the 6-port valve switched to allow the isocratic pump to wash away the residual salt solution in the RP-trapping column. The peptide-loaded trapping column was then switched to the RP-analytical column to start a new step of separation, and the bound peptides were eluted at a flow rate of 300 nl/min with a complete acetonitrile gradient (elution, regeneration, and then re-equilibration) in the presence of 0.1% formic acid over 65 min.

### Mass Spectrometry

The effluent of the online 2D LC system was analyzed by an Orbitrap Elite Hybrid Ion Trap-Orbitrap mass spectrometer (Thermo Electron, Bremen, Germany) equipped with a Nanospray Flex Ion Source. The instrument was operated in positive ion mode and the spray voltage was set to 1.8 kV. Full-scan MS spectra (m/z 400–m/z 2,000) were acquired in the Orbitrap mass analyzer at a resolution of 60,000 at m/z 400, and the MS/MS spectra were acquired in the linear ion trap (LTQ). The m/z 445.1200, 462.1466, and 536.1654 cyclosiloxane peaks were used for lock mass calibration in the Orbitrap to improve mass accuracy. The values of automatic gain control and the maximum accumulation times were 2 × 10^6^ and 1,000 ms for orbitrap, and 3,000 and 120 ms for LTQ analyzer, respectively. Acquisition of MS/MS spectra was done in a data-dependent manner. Fifteen most intense ions in each full-scan MS spectrum with a minimal signal intensity of 5,000 were selected for collision-induced fragmentation (CID) in LTQ with the following parameter settings: Isolation width of 2.0 Da, normalized collision energy of 35%, activation Q of 0.25, and activation time of 10 ms. Each precursor ion was allowed to be sequenced once and then excluded dynamically for 40 s.

### MS Data Analysis

The raw files were processed using the Proteome Discoverer software (version 1.3; Thermo Scientific, Waltham, MA). The CID spectra were searched against the UniProt database through Mascot search engine (version 2.2.2; Matrix Science Inc., Boston, MA). The search parameters were as follows: Enzyme specificity was trypsin; a maximum of two miscleavages were allowed; precursor mass tolerance was 10 ppm; fragment mass tolerance was 0.6 Da; ^13^C_6_ l-lysine, ^13^C_6_ l-arginine, acetylation at protein N-terminus, glutamine to pyroglutamic acid conversion at peptide N-terminus, and methionine oxidation were set as variable modifications; and cysteine carbamidomethylation was set as a fixed modification. False discovery rate (FDR) of peptide/protein identifications was determined by employing a decoy database searching. Peptide identifications were then filtered with the requirements of rank 1 peptides with a minimum peptide length of seven amino acid residues. Only high confident peptide/protein identifications (FDR < 1%) were considered for further evaluation.

Protein quantification was done with an SILAC workflow implemented in the Proteome Discover using the following parameters: Limits of mass and retention differences between heavy and light pairs should be smaller than 4 ppm and 0.2 min, respectively; only unique peptides were used for quantification; and protein ratio was determined using the median of the corresponding peptide ratios. Finally, functional annotation of each identified protein was retrieved from the ProteinCenter server (Thermo Scientific).

To investigate the proteomic effects caused by the dox treatment, we determined fold changes of SILAC signals upon dox treatment for all the identified proteins. First, the fold changes were indicated as signal ratios of heavy-/light-labeled peptides (H/L). For one protein identity, we considered the median H/L ratio to represent the fold change. Second, we normalized all of the fold changes with the median fold-change and log2-transformed the normalized fold change. Finally, we plotted the transformed data. To validate the reproducibility of the replicate SILAC experiment, we plotted fold changes of the two experiments against each other to calculate the Pearson’s correlation coefficient. We also considered the median SILAC signals (H/L for the first SILAC and L/H for the second) of untreated/treated cells to represent the fold change. We first log2-transformed the raw data of fold changes and then plotted the transformed data.

In the SILAC experiment, a protein was classified as misregulated (including up- and downregulated) in low-Hsp90 cells if its protein abundance was altered by more than 1.5-fold upon the dox treatment. Although this was an aribitrary cutoff, we also used a more stringent cutoff (>2-fold) to obtain another misregulated gene set and performed similar analyses. The results from the stringent cutoff still support our current conclusions. In addition, when we compared the overlap between two downregulated gene sets (1.5- and 2-fold) and previously identified Hsp90 interactor data, we found that these two downregulated sets had similar levels of overlap with the Hsp90 interactors (down-1.5-fold: 29.2%, down-2-fold: 34.5%, one-sided two-sample proportion test, *P* = 0.237), suggesting that using a 1.5-fold cutoff does not change the features of the data set radically.

### Microarray

Homemade oligonucleotide arrays were used (GPL7305). Total RNA was isolated using the Qiagen RNeasy Midi Kit (Qiagen, Valencia, CA). Complementary DNA of dox-treated and untreated cells was reverse transcribed with 15 μg RNA and labeled by SuperScript Indirect cDNA Labeling System (Invitrogen, Carlsbad, CA). Hybridization was performed with CodeLink Activated Slides (SurModics, Inc.). The array data were analyzed using GeneSpring GX (Agilent, Palio Alto, CA). The intensities for each array were normalized to Locally Weighted Scatterplot Smoothing (LOWESS) normalization (Cleveland 1981). Genes were classified as misregulated if the change in expression was larger than 1.5-fold with a *P* value smaller than 0.05.

### Pheromone Response and Mating Efficiency Assays

In order to measure the pheromone response, the *FUS1-* and *FUS3-GFP* strains were obtained from the yeast GFP collection (Huh et al. 2003). The strains were grown to log phase in 2× CSM medium and were treated with macbecin II (0, 3.125, 12.5, and 50 µM) for 1 h. Subsequently, the cells were exposed to 10 µM α-factor or vehicle dimethyl sulfoxide (as the control) for 2 h before being analyzed using BD FACSJazz machine (Becton-Dickinson Biosciences).

To measure the mating efficiency, log-phase a cells (S288C, *ho::HPH*) carrying a hygromycin B-resistant marker and α cells (S288C, *ho::KAN*) carrying a G418-resistant marker were treated with macbecin II (0, 12.5, and 50 µM) for 6 h. Subsequently, 2.5 × 10^6^ cells of each mating type were mixed, spread on a 0.22-µm nitrocellulose membrane and placed on YPD (Yeast Extract-Peptone-Dextrose) plates for mating. After mating for 1.5 h, cells were washed off, diluted, and plated on YPD plates containing hygromycin B at a density of about 300 colonies per plate. These colonies were then replica plated to different selective plates to distinguish their cell types. The mating efficiency was calculated by dividing the number of diploid cells by the number of cells carrying a hygromycin B-resistant marker.

### Evolutionary Rate Analysis

The coding sequences of *Saccharomyces cerevisiae* genes were retrieved in fasta format from Saccharomyces Genome Database (SGD, updated February 3, 2011). Pseudogenes and dubious open reading frames (ORFs) were excluded, yielding 5,888 total ORFs. For these *S. cerevisiae* genes, orthologs of *Candida glabrata* and *Kluyveromyces lactis* were obtained from the Fungal Orthogroup Repositiory (http://www.broadinstitute.org/regev/orthogroups/, last accessed October 20, 2014) (Byrne and Wolfe 2005; Wapinski et al. 2007). For evolutionary rate analyses in humans and four primate species, *Pan troglodytes*, *Gorilla gorilla*, *Pongo abelii*, and *Macaca mulatta*, all of the sequences including the orthologous gene pairs between human and primate species were downloaded from Ensembl Genome Browser (http://www.ensembl.org/index.html, last accessed October 20, 2014) (Release 74). To avoid ambiguous transcripts in human and primate species, we only used sequences that were also annotated in the Consensus CDS project (defined as CCDS genes). Furthermore, custom Perl scripts were used to scan pairwise alignments from MUSCLE (v3.8) (Edgar 2004) and identified the most similar splice variants of the human and primate protein orthologous sequence pairs. Our primary interest for these analyses was to determine the selection constraint on Hsp90-dependent proteins at a sequence level rather than the functional change in the proteins in the splice variants. Furthermore, to make a fair comparison between yeast and humans, it was necessary to compare the most similar splice forms, as yeast species of our interest generally lack splice variants.

The evolutionary rates (Ka/Ks) were computed by using CodeML program of the PAML (4.6) package (Yang 2007). For the analyses in yeasts, evolutionary rates were defined as the value of Ka/Ks between *S. cerevisiae* and *C. glabrata*; and the Ka value was used instead of Ka/Ks between *S. cerevisiae* and *K. lactis* to eliminate interference from the saturation of synonymous substitution (Ks) between the two species. For primates, the Ka/Ks was computed for the most similar orthologous splicing variants between human and primate protein-coding sequence (Matsuya et al. 2008; Georgi et al. 2013). From this analysis, we attempted to determine whether Hsp90-dependent proteins were under a selection constraint across species. The evolutionary rates of Hsp90-dependent proteins were tested against the evolutionary rates of the whole proteome background using one-sided Wilcoxon rank sum test, with the alternative hypothesis that Hsp90-dependent proteins had significantly lower evolutionary rates when compared with the whole proteome.

### Gene Ontology Analyses of Hsp90-Dependent Proteome

The gene ontology (GO) analyses were performed using FUNSPEC as described (Robinson et al. 2002) with Bonferroni correction and a *P* value threshold of less than 0.01. Enrichment score was calculated through dividing the proportion of Hsp90-dependent proteins classified into the indicated category by the proportion of those in the proteome (McClellan et al. 2007). The GO categories were deemed significant only if there was at least 2-fold enrichment and at least three ORFs were belonged to each GO term (Franzosa et al. 2011).

### Data Sets and Resources Used for the Study

The data used in this study were retrieved from public databases or from published research. The comprehensive list of *S. cerevisiae* transcription factors (TFs) was downloaded from ScerTF (http://stormo.wustl.edu/ScerTF/, last accessed October 20, 2014) (Spivak and Stormo 2012), the target ORFs of transcription factors were from Yeastract (http://www.yeastract.com/index.php, last accessed October 20, 2014) (Teixeira et al. 2014), protein molecular weight was from SGD (http://www.yeastgenome.org/, last accessed October 20, 2014), the unique physical and genetic interactions between proteins were from BioGRID (http://thebiogrid.org/, last accessed October 20, 2014) (Release 3.2.99), protein abundance was from the published research (Ghaemmaghami et al. 2003), the duplicate ORFs from small-scale duplication events were from the published research (VanderSluis et al. 2010), and the duplicate ORFs from whole-genome duplication were from YGOB (http://ygob.ucd.ie/, last accessed October 20, 2014). The final list of duplicate ORFs contained those from small-scale and whole-genome duplication. For essential ORFs for cell viability, data were from SGD (phenotype_data_25422). A comprehensive list of orthologous genes between *S. cerevisiae* and human was compiled by combining the available data sets from two databases, YeastMine (http://yeastmine.yeastgenome.org/yeastmine/begin.do, last accessed October 20, 2014) and HomoloGene (http://www.ncbi.nlm.nih.gov/homologene, last accessed October 20, 2014) (Release 67).

### Statistical Analyses

The statistical analyses were performed by R language (http://www.r-project.org/, last accessed October 20, 2014). One-sided Wilcoxon rank sum test was used to compute the statistic significance for the proteomic features, including evolutionary rates, protein half-lives, protein molecular weights, and unique interaction numbers. The effect size was computed in the R package coin. One-sided two-sample proportion test was for enrichment of the ORF with certain features, including essential ORFs, duplicate ORFs, and ORFs having human orthologs. Hypergeometric test was for enrichment of transcription factors and for that of GO terms with Bonferroni correction. The sample size, enrichment score, effect size, and *P* value of all statistical analyses are included in supplementary table S1, Supplementary Material online.

### Data Access

The MS proteomics data have been deposited to the ProteomeXchange Consortium (Vizcaino et al. 2014) through the PRIDE partner repository with the data set identifier PXD001162 and DOI 10.6019/PXD001162.

The microarray data are available at the GEO database (http://www.ncbi.nlm.nih.gov/geo/, last accessed October 20, 2014) under the accession number GSE56186.

## Results

### Hsp90 Maintains Protein Abundance Primarily through Posttranscriptional Regulation

Yeast cells contain two copies of Hsp90-encoding genes, the heat shock induced isoform *HSP82* and the constitutively expressed isoform *HSC82*, which are essential and redundant in function (Borkovich et al. 1989). As double deletion of both isoforms makes yeast cells inviable, we deleted *HSP82* and fused the *HSC82* coding region with a Tet-off promoter (Gari et al. 1997). By this strategy, the expression level of Hsp90 could be reduced significantly without disturbing cell viability when cells were grown in a medium containing 5 µg/ml of dox (Hsieh et al. 2013). The strain was further constructed to generate arginine and lysine double auxotrophs that also had a *CAR2* deleted to resolve the problem of arginine conversion in SILAC experiments (see Materials and Methods for details).

**Fig. 1.—:**
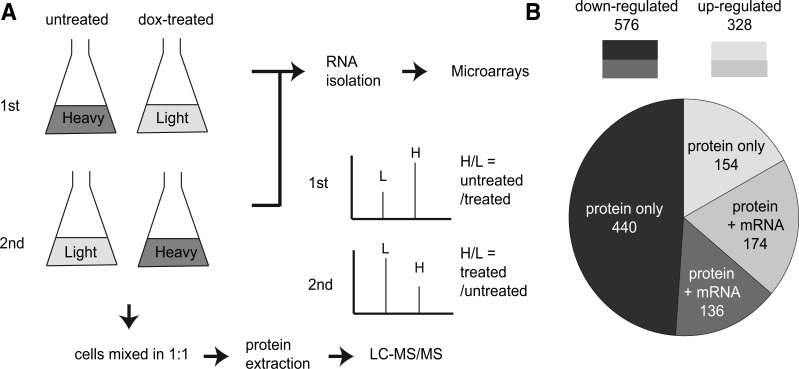
Overview of SILAC methodology and results. (*A*) Flow chart of the SILAC and microarray experiments. Cells were first grown in heavy or light amino acid-containing media. Before RNA and protein extraction, the experimental sets of cultures were treated with 5 μg/ml dox for 11 h to reduce the Hsp90 level. The culture was then split into two parts out of which one was used for RNA isolation. The RNA was processed to cDNA and subjected to microarray analyses. The remaining heavy- and light-labeled cells were combined in a 1:1 ratio of cell numbers and the total protein was extracted. The extracted total protein was in-solution digested and subjected to LC-MS/MS. The mass spectrum for the same peptide had paired signals (H vs. L) due to heavy isotope incorporation. Changes in the protein abundance upon dox treatments were inferred from the H/L ratio. (*B*) Hsp90 maintained protein abundance mainly through posttranscriptional regulation. “Protein only” denotes the ORFs that showed changes in protein abundance but not in the mRNA levels. “Protein + mRNA” denotes the ORFs that showed changes in both the protein and mRNA levels.

The resulting strain was subjected to label-swap replication of SILAC coupled with LC–MS/MS for relative protein quantification. In the first SILAC experiment, the light amino acid-labeled cells were treated with dox to reduce the Hsp90 level and the heavy amino acid-labeled cells served as a wild-type control. After labeling, the light- and heavy-labeled cells were mixed in a 1:1 ratio in cell numbers and the proteomic difference between these two populations was measured using H/L ratios resolved by MS (fig. 1*A*). In the second experiment, the treatment was swapped to control for any potential labeling effect. To determine whether the effect of Hsp90 on the proteome was transcriptional or posttranscriptional, we simultaneously collected total RNA from the same populations of cells and measured changes in mRNA expression by microarrays.

The SILAC data were first filtered to remove the low-confident results, which were proteins identified by less than two unique peptides. After filtering, we identified 4,041 proteins in the first SILAC experiment and 3,696 proteins in the second experiment. Neither the dox treatment nor labeling exhibited a global effect on protein abundance. Majority of the proteins showed no difference in abundance after the dox treatment in both SILAC experiments (supplementary fig. S1, Supplementary Material online). When two SILAC results were compared, 3,561 proteins were identified in both experiments. Moreover, a strong correlation was observed in the detected protein changes between two experiments (Pearson’s coefficient *r* = 0.896, *P* < 2.2 × 10^−^^16^), suggesting that different labelings had no influence on the pooled SILAC experiments and the observed results were reproducible (supplementary fig. S2, Supplementary Material online). We decided to pool these two sets of data together for further analyses. The total coverage of the SILAC-determined proteins reached 72% of the yeast proteome and is close to the total number of proteins expressed under standard growth conditions (Nagaraj et al. 2012).

In this study, a protein was considered misregulated (including up- and downregulated) in low-Hsp90 cells if its protein abundance was altered by more than 1.5-fold upon dox treatment. We found that 904 proteins were misregulated in low-Hsp90 cells (supplementary table S2 and fig. S3, Supplementary Material online). When the data were compared with the mRNA expression data from microarrays, 66% of them (594/904 = 66%) exhibited no obvious changes at the mRNA level (more than 1.5-fold with a *P* value smaller than 0.05). These results suggest that majority of the misregulated proteins were influenced by Hsp90 posttranscriptionally. Among this group of proteins, most of them (440 of 594) were downregulated, suggesting that translation or protein stability of these proteins depends on Hsp90 (fig. 1*B*).

To further confirm the SILAC results, we selected nine genes among the downregulated group and examined their protein abundance using GFP fusion protein constructs. The cells were grown in medium with or without the Hsp90 inhibitor macbecin II, and then GFP fusion protein levels were measured using a fluorescence-activated cell sorter (FACS). The fold change of these proteins displayed a consistent pattern between the FACS and SILAC data (Pearson’s coefficient *r* = 0.835, *P* = 0.005; supplementary fig. S4, Supplementary Material online).

### The Downregulated Proteome of Low-Hsp90 Cells Is Conserved and Enriched in Essential Proteins

**Fig. 2.—:**
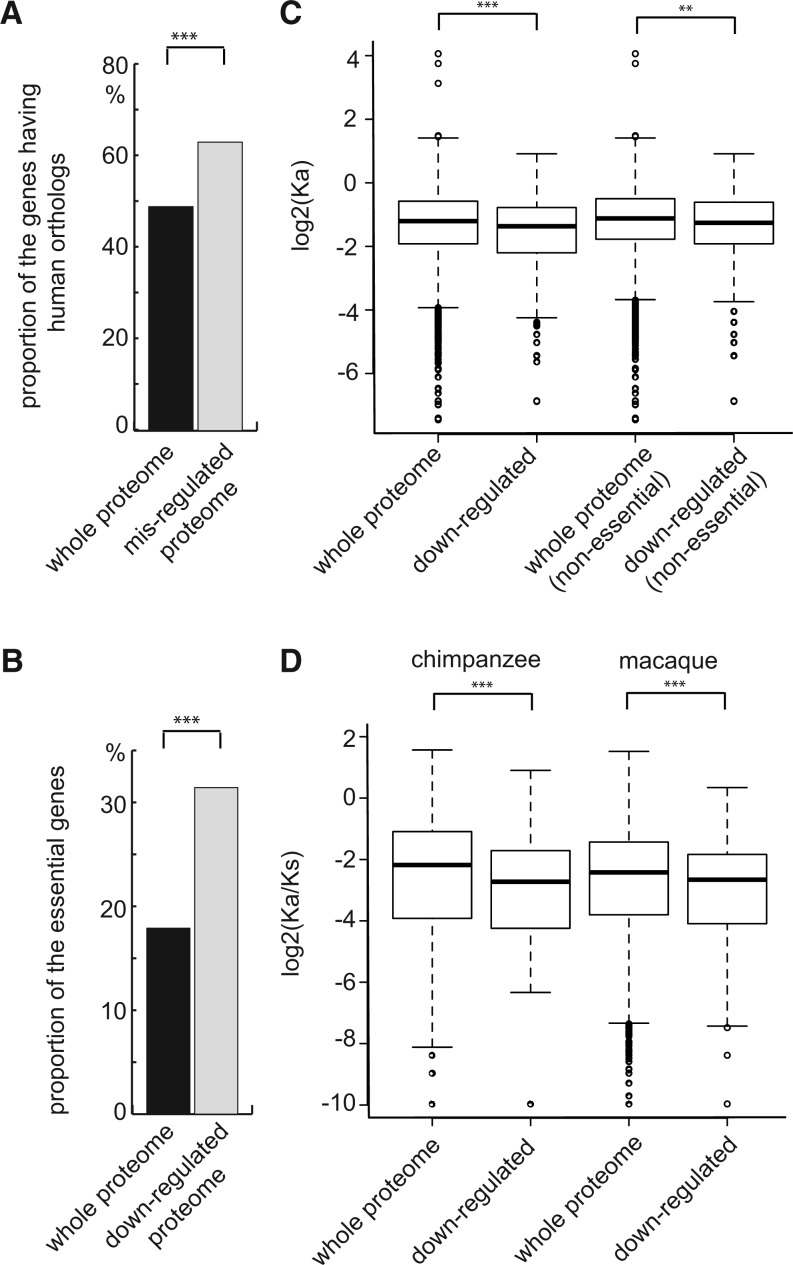
The downregulated ORFs are highly conserved and enriched in essential genes. (*A*) The misregulated proteome of low-Hsp90 cells has a higher proportion of ORFs having human orthologs compared with the whole proteome (one-sided two sample proportion test, *P* = 1.82 × 10^−10^). (*B*) The proportion of essential genes in the downregulated group is higher compared with the whole proteome background (one-sided two sample proportion test, *P* = 1.74 × 10^−5^). (*C*) Analysis using orthologs between two distant yeast species, *S. cerevisiae* and *K. lactis*, shows that the evolutionary rate is lower in the downregulated group than in the whole proteome. The evolutionary rate of the downregulated group was observed to be consistently lower than the whole proteome background even when only nonessential genes were compared. Similar conclusions were obtained from another analysis using two more closely related yeast species (shown in supplementary fig. S4, Supplementary Material online). Nonsynonymous substitution rate (Ka) instead of the nonsynonymous to synonymous substitution ratio (Ka/Ks) was used because the number of synonymous mutations is likely to be saturated between two distant species and it can lead to severe bias in evolutionary rates. (*D*) The downregulated proteins of human low-Hsp90 cells also exhibited low evolutionary rates. Orthologs between humans and indicated primates were analyzed, and Ka/Ks was used in this analysis as the divergence times between primates were quite recent. **P < 0.01, ***P < 0.001.

Hsp90 is a highly conserved molecular chaperone and can be found from yeast to humans (Chang and Lindquist 1994). Human Hsp90 was able to complement defects caused by deletion of its counterpart in yeast cells (Picard et al. 1990; Millson et al. 2007). It is interesting to know whether the misregulated proteins discovered in our SILAC experiments are more likely to have human orthologs. Our analysis showed that indeed a large proportion of the misregulated proteins (572/904 = 63%) have human orthologs compared with the whole proteome background (3,209/6,542 = 49%) (one-sided two-sample proportion test, enrichment score = 1.29, *P* = 1.82 × 10^−^^10^) (fig. 2*A*). We also observed that the downregulated proteins of low-Hsp90 cells were enriched in proteins encoded by essential genes (31% in the downregulated group compared with 18% in the whole proteome background; one-sided two-sample proportion test, enrichment score = 1.74, *P* = 1.74 × 10^−^^5^; fig. 2*B*). These results suggest that the inviability of Hsp90 deletion mutants may result from the deficiency of Hsp90-dependent essential genes.

It has long been speculated that molecular chaperones are able to accelerate the evolutionary rate of their clients under the direct buffering hypothesis (Bogumil and Dagan 2010; Lachowiec et al. 2013). We tested this idea by estimating the evolutionary rates (using the nucleotide substitution rate as a proxy) between orthologs of *S. cerevisiae* and other two yeast species from the pre- and post-whole-genome duplication clades namely *K. lactis* and *C. glabrata* (supplementary table S3, Supplementary Material online). Surprisingly, the evolutionary rates of downregulated proteins derived from the pairwise comparisons of *S. cerevisiae* with both the abovementioned species were significantly lower than that of the whole proteome of *S. cerevisiae* (one-sided Wilcoxon rank-sum test, *P* = 1.33 × 10^−^^5^ *S. cerevisiae* vs. *K. lactis*; *P* = 2.21 × 10^−^^5^ *S. cerevisiae* vs. *C. glabrata*; fig. 2*C* and supplementary fig. S5*A*, Supplementary Material online).

As essential genes tend to be conserved, it is possible that the observed low evolutionary rates are due to the fact that the downregulated protein list contains many proteins encoded by essential genes. We removed the essential genes from the list and assessed the sequence conservation of nonessential genes. The result shows that the evolutionary rate remained consistently lower than the whole proteome background even when only nonessential genes were compared (one-sided Wilcoxon rank-sum test, *P* = 9.3 × 10^−^^3^ *S. cerevisiae* vs. *K. lactis*; *P* = 0.07 *S. cerevisiae* vs. *C. glabrata*; fig. 2*C* and supplementary fig. S5*A*, Supplementary Material online). The same trend was also observed for the upregulated proteins (supplementary fig. S5*B*, Supplementary Material online).

To investigate whether the high sequence conservation only represents a yeast-specific phenomenon, we used the data from a previous human Hsp90-dependent proteome study to perform similar analyses (Sharma et al. 2012). A low evolutionary rate was also observed in human Hsp90-dependent proteome when compared orthologs between humans and other primates (fig. 2*D*; supplementary table S3 and fig. S5*C*, Supplementary Material online). Taken together, our results suggest that the Hsp90-dependent proteome evolves slowly and tends to be conserved.

### The Downregulated Proteins in Low-Hsp90 Cells Have High Connectivity

**Fig. 3.—:**
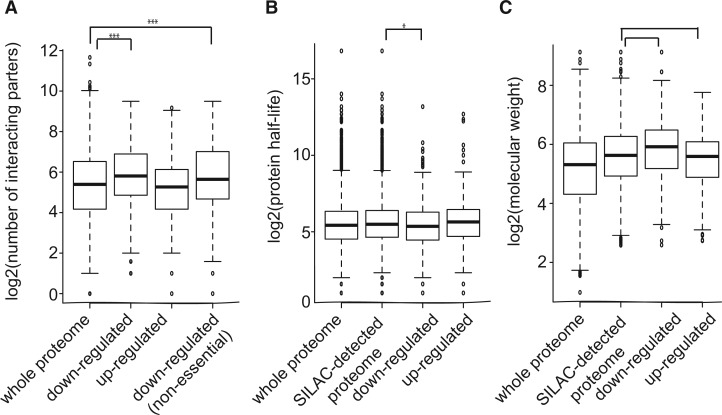
The downregulated proteome of low-Hsp90 cells has high connectivity and molecular weight. (*A*) Proteins in the downregulated group have a high unique interaction number. This trend was observed even after the removal of proteins encoded by essential genes from this group. (*B*) The protein half-life of the down group was less than the SILAC-detected proteome background. (*C*) The molecular weight of proteins in the down group was observed to be larger than that of the SILAC-detected proteome. *P < 0.05, ***P < 0.001.

Proteins with more protein–protein interactions have been shown to evolve more slowly and have important functions in the cell (Fraser et al. 2002). Moreover, it has been suggested that network hubs (highly connected nodes in a protein–protein interaction network) often have the ability to buffer genetic or environmental variations (Bergman and Siegal 2003; Levy and Siegal 2008). We investigated whether the Hsp90-dependent proteins also have a higher number of interactors. Using the BioGRID database (Stark et al. 2006), we found that the unique interaction number of the downregulated proteins was significantly higher (median = 55) than that of the whole proteome (median = 42) (one-sided Wilcoxon rank-sum test, *P* = 1.92 × 10^−^^9^; fig. 3*A*).

Proteins encoded by essential genes have been reported to have more protein–protein interactions (Jeong et al. 2001). To rule out the possibility that the high interaction number observed in the downregulated proteome was simply due to the enrichment of proteins encoded by essential genes in the list, we analyzed nonessential downregulated proteins separately. The result showed that nonessential downregulated proteins still had a higher interaction number (median = 48) compared with the whole proteome (one-sided Wilcoxon rank-sum test, *P* = 9.91 × 10^−^^5^; fig. 3*A*). Overall, these observations provide evidence to demonstrate that other than Hsp90 itself, many downregulated proteins in low-Hsp90 cells also act as hubs. This observation offers an alternative mechanism by which Hsp90 can buffer both genetic and nongenetic variations in various cellular processes (discussed below).

### The Downregulated Proteins Are Unstable and Have High Molecular Weights

We have observed that the downregulated proteins in low-Hsp90 cells tend to be more conserved and form protein network hubs. We next asked what are the physicochemical properties shared by this group of proteins. Thermal stability has been found to be an important determinant for some Hsp90 interacting kinases (Taipale et al. 2012). Because unstable proteins usually have short protein half-lives, we investigated whether the yeast Hsp90-dependent proteins displayed similar features. Given that highly unstable and/or small proteins are hard to detect by MS, we used the SILAC-detected proteome as the background for comparison, instead of the whole yeast proteome. Using experimental data of protein half-lives in yeast (Belle et al. 2006), we found that the downregulated proteins exhibited a shorter protein half-life (median = 41 min) than the background (median = 45 min) (one-sided Wilcoxon rank-sum test, *P* = 0.01; fig. 3*B*). In contrast, the half-life of the upregulated proteins did not show a significant difference compared with that of the background.

In general, large proteins with multiple domains are more likely to have high interaction partners. In addition, larger proteins may be less stable and require more help from molecular chaperones for their folding. We tested whether that was the case for the downregulated proteins of low-Hsp90 cells. Our analyses found that the molecular weight of the downregulated proteins (median = 60.1 kDa) was significantly higher than that of the background (median = 49.4 kDa) (one-sided Wilcoxon rank-sum test, *P* = 1.28 × 10^−^^9^; fig. 3*C*). In contrast, the upregulated proteins had lower molecular weights (median = 47.8 kDa) compared with background. A previous study in the human Hsp90-dependent proteome revealed a similar trend (Sharma et al. 2012). These results suggest that high molecular weight is a common property of the downregulated proteins in low-Hsp90 cells.

### Hsp90 Reduction Affects Proteins that Perform Major Cellular Functions

**Fig. 4.—:**
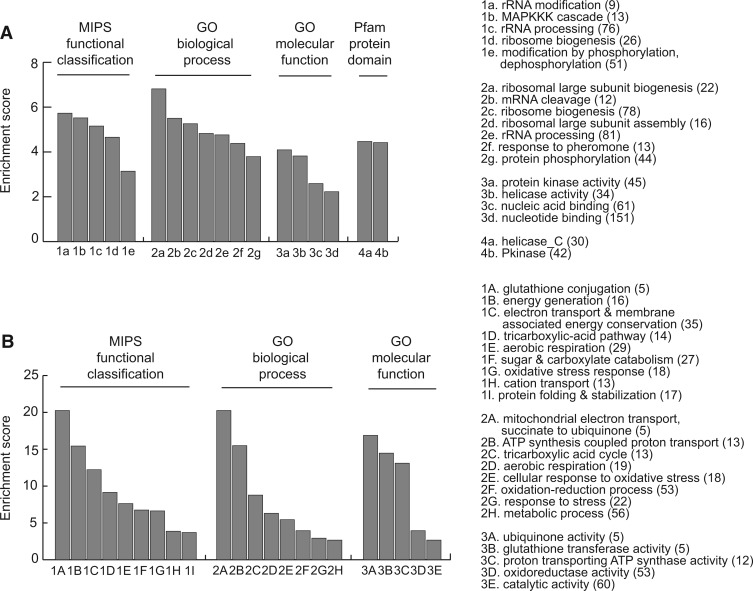
Analyses for the molecular functions and protein domains of the down- and upregulated proteins in low-Hsp90 cells. Hsp90-dependent proteins were analyzed using MIPS for functional classification, GO for biological process and molecular function, and Pfam database for protein domains. The enriched categories of down- and upregulated proteins are shown in (*A*) and (*B*), respectively. Enrichment score was calculated through dividing the proportion of Hsp90-dependent proteins classified into the indicated category by the proportion of those in the proteome. Numbers in the parenthesis indicate the number of Hsp90-dependent proteins classified into the indicated categories. All of the categories have corrected *P* values of less than 0.01.

After examining the general features of Hsp90-dependent proteome, we next determined the pathways or functions in which those proteins are enriched using the integrated database FunSpec (Robinson et al. 2002). We first focused on the downregulated proteins. Ranking the Munich Information Center for Protein Sequences (MIPS) and GO results based on the enrichment score revealed a few interesting categories as shown in figure 4*A* and supplementary table S4, Supplementary Material online. Many of them (e.g., phosphorylation, rRNA processing, and ribosome biogenesis) have been observed in previous studies using different methods, suggesting that the SILAC method is a useful approach for identifying specific Hsp90-dependent pathways or functions (McClellan et al. 2007; Zhao et al. 2008). In addition, our analyses detected some specific processes such as “MAPKKK cascade,” “mRNA cleavage,” and “DNA repair” which have not been reported in earlier genome-wide studies of yeast Hsp90 (supplementary table S4, Supplementary Material online).

When the protein domains of the downregulated proteins were analyzed, they were enriched in the protein kinase and helicase conserved C-terminal domains (fig. 4*A*). The enrichment of protein kinase domains in Hsp90-dependent proteins was not surprising as Hsp90 is known to influence many signal transduction pathways containing protein kinases (Taipale et al. 2010). On the other hand, the enrichment of the helicase domain is less understood. Thus, we decided to analyze them further. We found that the downregulated proteins with the helicase conserved C-terminal domain were specifically enriched in the “DNA repair” category (hypergeometric test, enrichment score = 4.47, corrected *P = *0.02), which has also been identified in our analysis of the functional classification.

Many transcription factors have been observed to be the targets of Hsp90 in previous studies (Csermely et al. 1998; Taipale et al. 2010). Although in our downregulated protein list, the category of transcriptional control was not highly enriched, we performed a closer inspection of transcription factors. A comprehensive list of transcription factors was retrieved from the ScerTF database (Spivak and Stormo 2012), which covers 196 experimentally validated transcription factors from 11 different sources. Around 11% (21 of 196) of these yeast transcription factors were found in our posttranscriptionally downregulated proteome and they were significantly enriched (hypergeometric test, enrichment score = 1.61, *P* = 0.01). Moreover, 15 of these 21 transcription factors have not been known to be Hsp90-dependent previously (Stark et al. 2006) (supplementary table S2, Supplementary Material online).

In the upregulated proteome, we observed many cochaperones, small HSPs, and stress response proteins that led to enrichments in the categories of “stress response,” “protein folding and stabilization,” and “unfolded protein response” (fig. 4*B*). It is likely that these proteins were upregulated to compensate the effect caused by the reduction of Hsp90. Other significant categories include “mitochondrial electron transport,” “oxidative stress,” and “metabolic processes.” Some of these categories have been observed previously (Morano et al. 2012; Sharma et al. 2012; Wu et al. 2012).

### Downregulation of Hsp90-Dependent Proteins Leads to Physiological Changes of the Cell

Pathway analyses have revealed that Hsp90-dependent proteins are involved in many important cellular processes. We further experimentally examined whether downregulation of the Hsp90-dependent proteins leads to detectable phenotypic changes of the cell. Because the downstream target genes of many transcription factors have been identified and experimentally validated (Teixeira et al. 2014), it allowed us to check the effect of the downregulated transcription factors on their direct target genes using our microarray data. From the YEASTRACT database, we found 12 transcription factors in our downregulated list that have well-characterized target gene information. When the expression levels of those target genes in low-Hsp90 cells were analyzed, it revealed that eight transcription factors showed obvious effects on their target genes, under the criterion that more than two target genes of an individual transcriptional factor have changed their mRNA levels significantly (table 1).

**Table 1:** Downregulation of Transcription Factors in Low-Hsp90 Cells Shows Obvious Effects on Their Target Genes

Transcription Factor^a^	Target Gene^b^
Aft1	*ATH1, FRE5, REC114, NMD3, YHR138C*
Bas1	*ADE2, ADE8, ADE13, ADE17, CAT2, DAS2, FLC2, GCV1, GCV2, HOP1, MTD1, OAC1, QCR9, SHM2, SNO3, SNZ3, YBLO29W, YDR018C*
Hap1	*CTY1, OLE1, PET10, RIP1*
Ino4	*CHO1, IAH1, ITR1, SPL2, YIRO42C, YOL107W*
Leu3	*CDA1, GDH1, ILV3*
Met4	*CWP1, MET2, MET3, MET6, MET8, MET14, MUP1, PCL5, SPL2, YGP1, ZWF1, YHRO33W*
Oaf1	*ARG80, CTA1, CUP9, GLC3, YDR344C*
Rds2	*ATP2, ISF1, PTR2, TIP1, UTH1, ZEO1*

^a^Only the transcription factors of which more than two target genes have changed their mRNA levels significantly in low-Hsp90 cells are shown in the table.

^b^The expression patterns of the target genes in low-Hsp90 cells are consistent with previous experimental results shown in low or mutant TF cells.

**Fig. 5.—:**
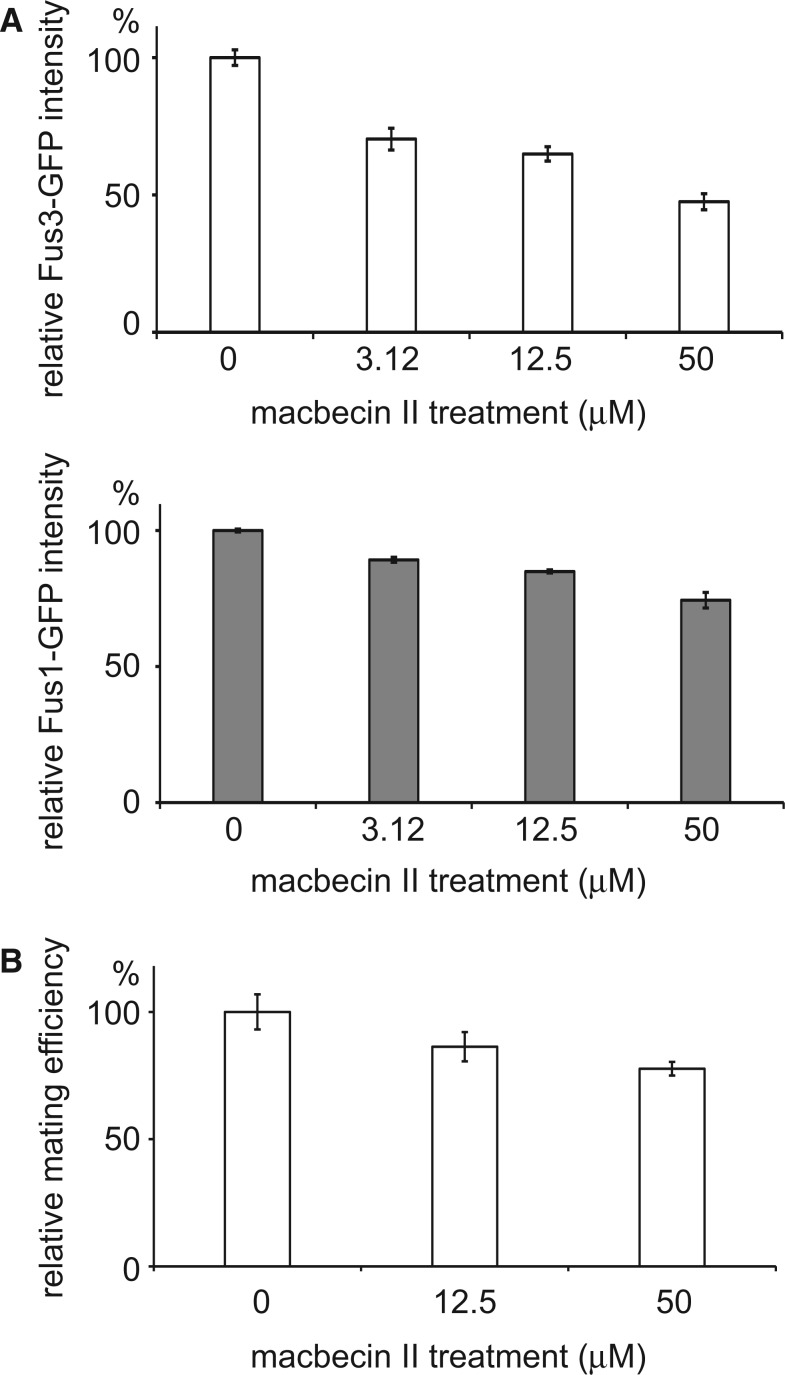
Downregulation of the Hsp90 activity causes defects in the yeast mating pathway. (*A*) Fus3 is an MAPKKK that is downregulated in low-Hsp90 cells and *FUS1* is a downstream gene of the mating pathway. Cells carrying Fus3-GFP or Fus1-GFP were treated with different concentrations of the Hsp90 inhibitor macbecin II and then subjected to the mating pheromone to activate the mating pathway. The GFP signal was measured using the FACS. All signal intensities were normalized to that of untreated controls. Error bars represent the standard error from three independent repeats. (*B*) Haploid cells with reduced Hsp90 activity mate less efficiently. a and α cells were treated with different concentrations of macbecin II and then assayed for their mating efficiency. The obtained mating efficiencies were normalized to that of untreated controls. Error bars represent the standard error from six independent repeats.

The yeast mating pathway contains a mitogen-activated protein kinase (MAPK) cascade in which three components (Fus3, Ste7, and Ste5) are downregulated in low-Hsp90 cells. A previous study has shown that the mating pathway is defective in Hsp90 mutant cells (Louvion et al. 1998). We investigated how the mating pathway was influenced by different levels of the Hsp90 activity. Two GFP-tagged yeast strains were used in which GFP was fused with the Hsp90-dependent gene, *FUS3*, or a downstream gene of the MAPK cascade, *FUS1* (Huh et al. 2003). Fus3-GFP was used to monitor the levels of the Hsp90 activity and Fus1-GFP was used to measure the activity of the mating pathway. When the GFP fusion protein carrying cells were treated with increasing concentrations of macbecin II, the Fus3-GFP and Fus1-GFP levels were reduced in response to α-factor in the inhibitor-treated samples, indicating that the pathway is sensitive to the activity level of Hsp90 (fig. 5*A*). Finally, we performed a mating efficiency assay and showed that macbecin II-treated cells indeed mated less efficiently (fig. 5*B*). Our experiments in the Hsp90-dependent transcription factors and MAPK cascade suggest that downregulation of these proteins has impacts on cell physiology.

## Discussion

Hsp90 is a chaperone that assists maturation of many regulators involved in different cellular processes and repairs misfolded proteins (Taipale et al. 2010; Trepel et al. 2010). Although earlier studies in yeast provide considerable information on the physical and genetic interactomes of Hsp90, our understanding of how different Hsp90 interactors are influenced leading to the observed phenotypes when Hsp90 is compromised is still limited. In our present study, we investigated the effect of compromised Hsp90 on both the proteome and transcriptome using the same yeast populations. In total, we identified 904 misregulated proteins in low-Hsp90 cells. Two-thirds of them (594 of 904) do not show changes in their mRNA levels, suggesting that the majority of the Hsp90-dependent proteins we identified are regulated posttranscriptionally.

Many Hsp90 interactors have been shown to become unstable when their association with Hsp90 was disturbed by an Hsp90 inhibitor (Taipale et al. 2012). Interestingly, among 440 posttranscriptionally downregulated proteins identified in our experiments, only 123 of them were previously curated as Hsp90 interactors (Stark et al. 2006). It is likely that not all Hsp90 interactors are subjected to the same level of influence in low-Hsp90 cells. On the other hand, we often observed multiple components from the same pathways in our downregulated protein list. It has long been speculated and recently shown in a few studies that extra copies of the subunits in some protein complexes would be degraded in order to maintain normal protein complex stoichiometry in the cell (Goldberg and Dice 1974; Geiger et al. 2010; Stingele et al. 2012). This phenomenon may explain our observation that multiple components of the same pathway were downregulated in low-Hsp90 cells even if only one of them is an Hsp90 interactor. In our function and pathway enrichment analysis, other than regulatory proteins (e.g., kinases, TFs, etc.) that many Hsp90 studies have focused on previously, we also observed strong enrichments for other functions and processes such as ribosome biogenesis, assembly, and mRNA cleavage. We need to understand how Hsp90 influences these processes before we can construct a complete regulatory network of Hsp90.

In eukaryotes, impairment of Hsp90 has been found to reveal cryptic genetic variation in organisms ranging from yeast to vertebrates to plants (Jarosz et al. 2010; Rohner et al. 2013). Recently, it has also been demonstrated that compromising Hsp90 activity increases developmental and morphological noise in plants and yeast (Queitsch et al. 2002; Sangster et al. 2007; Hsieh et al. 2013). The function of Hsp90 in buffering diverse genetic and nongenetic variations suggests that Hsp90 is important for maintaining phenotypic robustness of cells when facing genetic or environmental perturbations. Phenotypic robustness has been implicated in shaping short-term population adaptation or long-term organism evolution (Fraser and Kaern 2009; Masel and Siegal 2009). However, the nature of Hsp90 buffering remains mysterious in most reported cases. Our analyses of the Hsp90-dependent proteome provide a direct link between Hsp90 buffering and previously identified features of a robust system.

The downregulated proteins in low-Hsp90 cells are observed to interact with a large number of other proteins even after the removal of proteins encoded by essential genes from the list (fig. 3*A*). Making highly connected networks could be an efficient way to confer robustness to a system, where multiple layers of regulation can work together to achieve better control (Albert et al. 2000). Studies in computational simulation and yeast experiments have also provided evidence that compromising network hubs allows cells to reveal cryptic genetic variation or population heterogeneity (Bergman and Siegal 2003; Levy and Siegal 2008). By regulating the stability of hub proteins, Hsp90 is able to buffer their downstream genes in various pathways. Unlike the direct buffering model, which predicts that Hsp90 only buffers mutations causing protein-folding defects (Fares et al. 2002; Sangster et al. 2004), indirect buffering (through network hubs) allows cells to accumulate various types of mutations.

Further evidence supporting the indirect buffering model of Hsp90 comes from the evolutionary rate analyses of Hsp90-dependent proteins. If Hsp90 directly buffers genetic variation, the ensuing robustness would allow its targets to accumulate more mutations resulting in high evolutionary rates. As shown in previous studies of bacterial chaperones GroEL/GroES, accelerated evolutionary rates are observed in GroEL-dependent proteins in both experimentally evolved populations and natural species (Tokuriki and Tawfik 2009; Bogumil and Dagan 2010). However, when the evolutionary rate of the downregulated proteins in low-Hsp90 cells was examined, we observed the opposite trend in both yeast and human cells. This trend persists even in the downregulated nonessential proteins, suggesting that the constraint is not because of essentiality. These results suggest that Hsp90 may allow mutation accumulation in the secondary or further downstream interactors, instead of its primary targets. When the buffering effect is mediated through those conserved Hsp90-dependent hubs, it allows organisms to reveal the effect of different mutations depending on their genetic backgrounds or environmental challenges. It increases the flexibility of the Hsp90 buffering system and may also explain how Hsp90 operates on roughly 20% of the pre-existing genetic variation in *S. cerevisiae* populations (Jarosz and Lindquist 2010).

Why do Hsp90-dependent proteins tend to be network hubs? A larger protein comprising multiple interacting domains will have a higher chance to evolve high connectivity. However, the tradeoff would be that such proteins may have to undergo more complicated protein folding and display lower stability (Bogumil and Dagan 2012). It is possible that the chaperone activity of Hsp90 helps large and unstable proteins to be maintained in the cell and eventually allows them to evolve into network hubs. Consistent with this hypothesis, we find that the downregulated proteins are relatively unstable and have relatively higher molecular weights (fig. 3*B* and *C*).

The Hsp90-dependent proteome we have identified is functionally conserved between yeast and human cells. GO analyses show that the yeast Hsp90-regulated proteome has a general consensus with its human counterpart, where the major effects are the downregulation of kinases and the upregulation of unfolded response proteins (Sharma et al. 2012; Wu et al. 2012). In addition, the Hsp90-dependent proteins identified in this work also provide novel candidates for future phenotypic studies. In our downregulated protein list, we identified 25 protein kinases and 15 transcription factors previously unknown to be regulated by Hsp90 in yeast. Examining the effects of decreases in these kinases and transcription factors will help us dissect the underlying mechanism of observed phenotypes caused by compromised Hsp90. Moreover, our results reveal that the nature of buffering and release of variation by Hsp90 is not deviated from general features of a robust system in which constant phenotypes are achieved or disrupted by regulating network hubs. The network formed by Hsp90-dependent proteins will provide us the starting materials for uncovering the buffering capacity and evolutionary influence of Hsp90.

## Supplementary Material

Supplementary figures S1–S5 and tables S1–S4 are available at *Genome Biology and Evolution* online (http://www.gbe.oxfordjournals.org/).

Supplementary Data
