# Supplementary material for: The Hsp90-Dependent Proteome Is Conserved and Enriched for Hub Proteins with High Levels of Protein–Protein Connectivity
Source: Genome Biol Evol. 2014 Oct 13;6(10):2851–65. doi: 10.1093/gbe/evu226 (PMC4224352; doi:10.1093/gbe/evu226)
Supplement: Supplementary Data [file supp_6_10_2851__index.html]

The Hsp90-dependent proteome is conserved and enriched for hub proteins with high levels of protein-protein connectivity — The Hsp90-Dependent Proteome Is Conserved and Enriched for Hub Proteins with High Levels of Protein–Protein Connectivity — Supplementary Data 

# The Hsp90-Dependent Proteome Is Conserved and Enriched for Hub Proteins with High Levels of Protein–Protein Connectivity

## Supplementary Data

files

**Files in this Data Supplement:**

- Supplementary Data - pdf file
- Supplementary Data - docx file
- Supplementary Data - docx file
- Supplementary Data - xlsx file
- Supplementary Data - doc file
- Supplementary Data - xlsx file
