# Supplementary figures and images for: The Hsp90-Dependent Proteome Is Conserved and Enriched for Hub Proteins with High Levels of Protein–Protein Connectivity
Source: Genome Biol Evol. 2014 Oct 13;6(10):2851–65. doi: 10.1093/gbe/evu226 (PMC4224352; doi:10.1093/gbe/evu226)

Figure S1

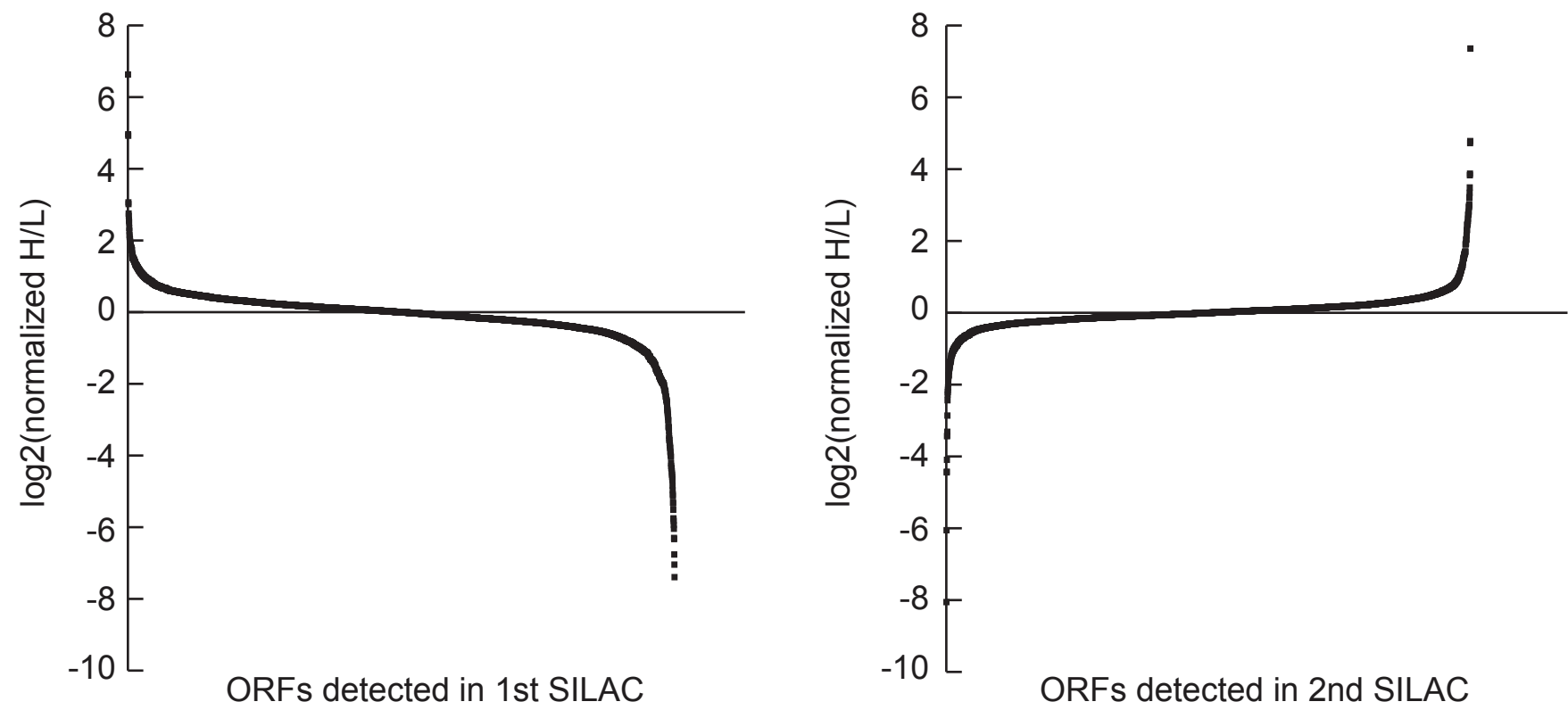

Figure S2

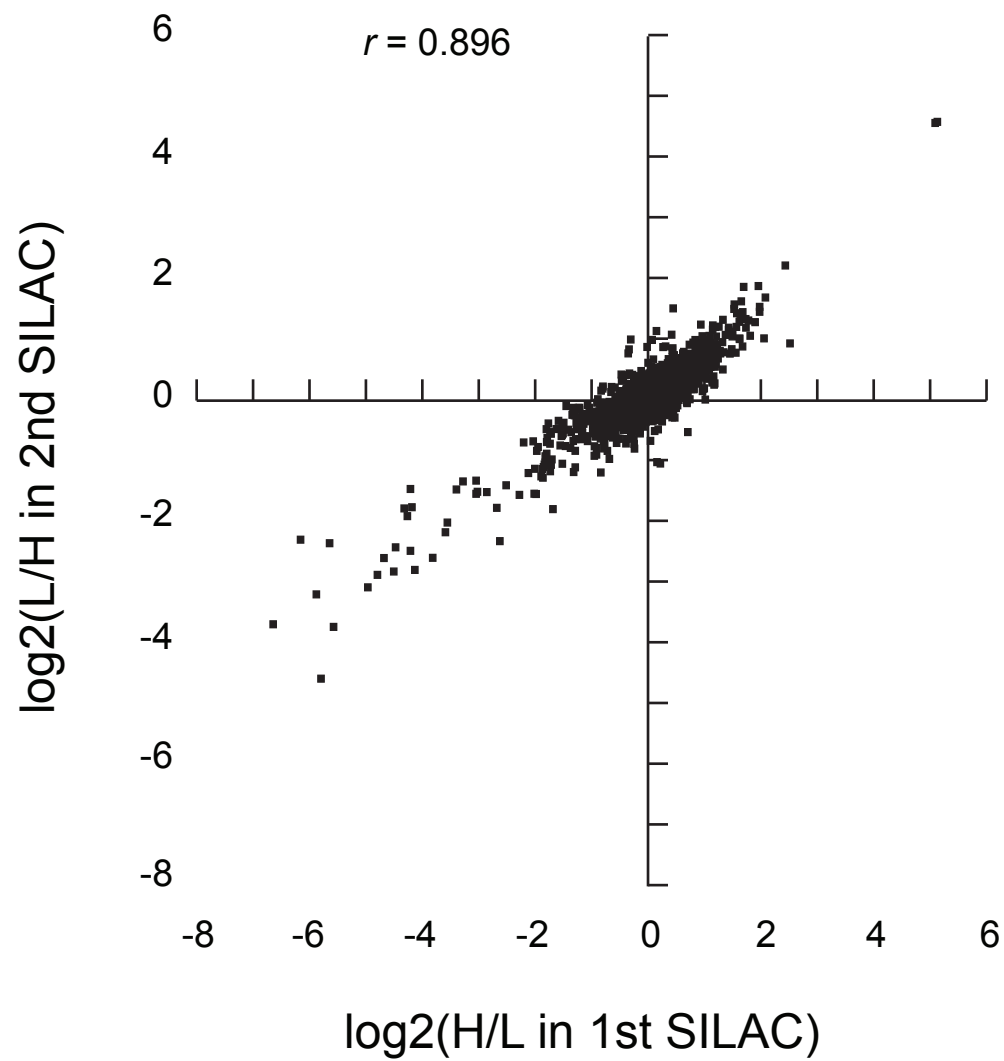

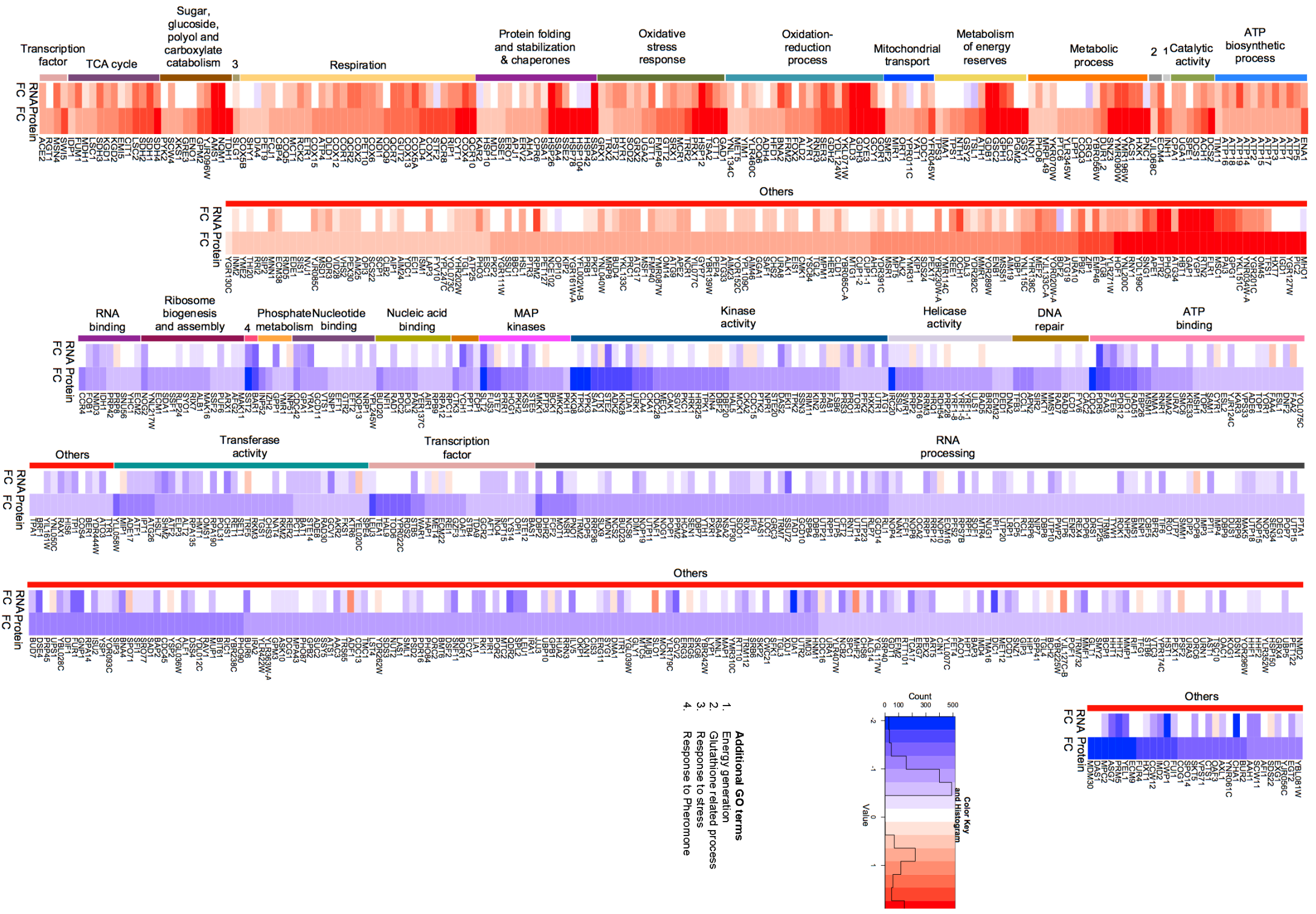

Figure S4

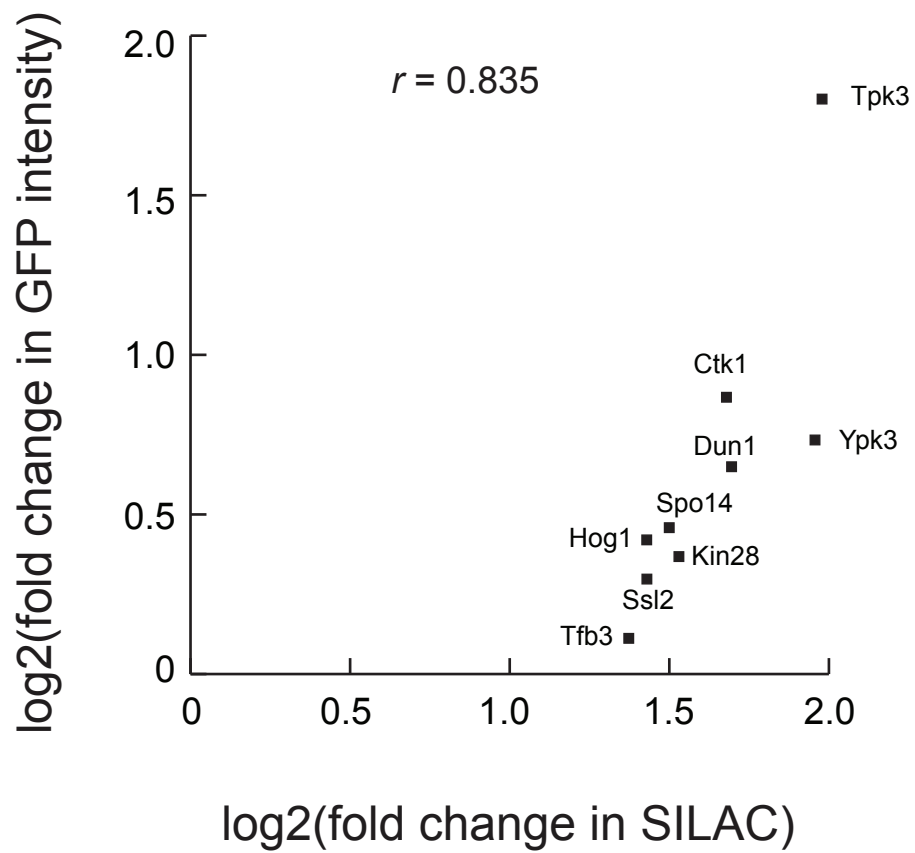

Figure S5

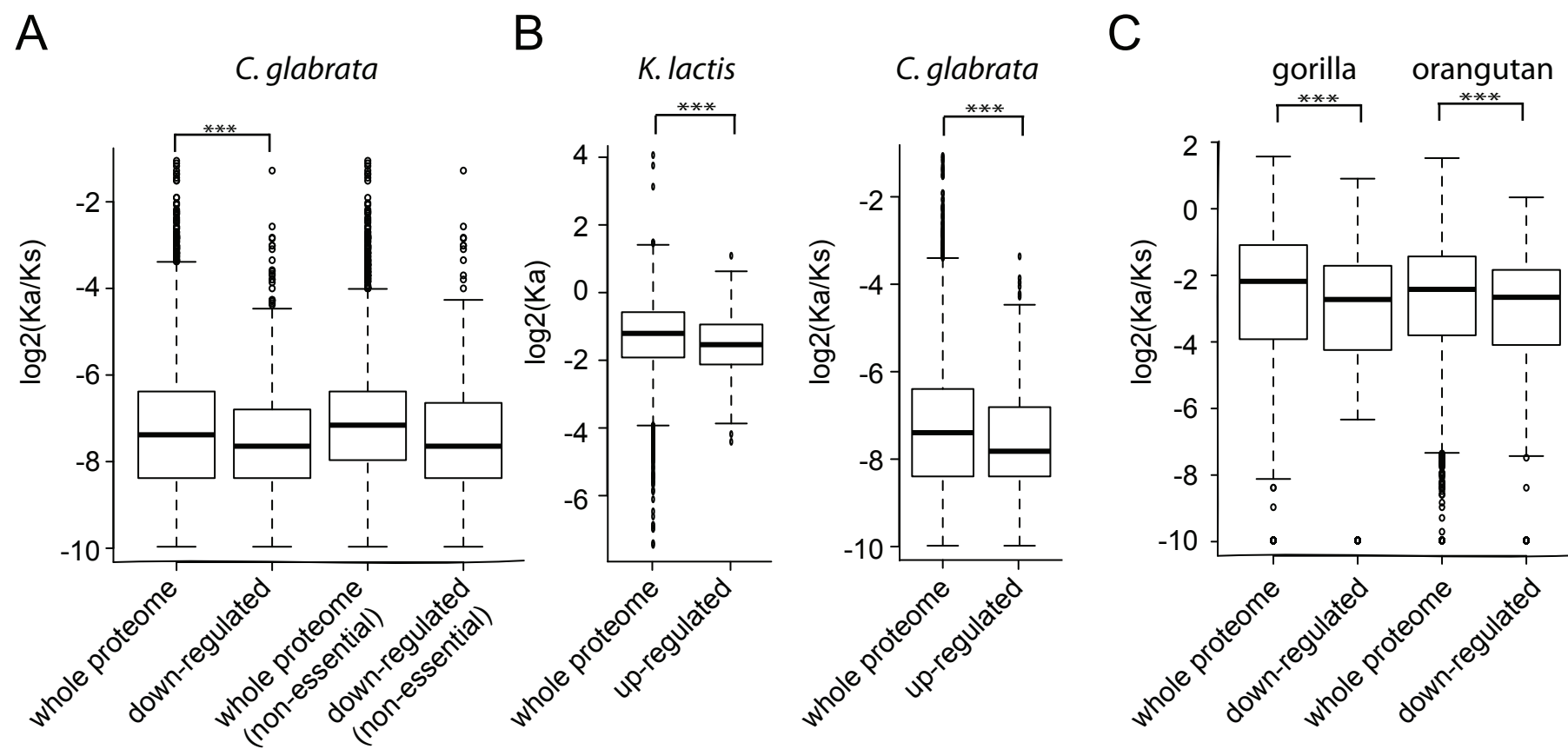

Supplement: Supplementary Data [file supp_evu226_Supp_Fig.pdf]
