# Supplementary material for: The Hsp90-Dependent Proteome Is Conserved and Enriched for Hub Proteins with High Levels of Protein–Protein Connectivity
Source: Genome Biol Evol. 2014 Oct 13;6(10):2851–65. doi: 10.1093/gbe/evu226 (PMC4224352; doi:10.1093/gbe/evu226)
Supplement: Supplementary Data [file supp_evu226_New_Microsoft_Office_Word_Document.docx]

**Supplemental Materials**

**Supplemental Figure Legends**

**Figure S1** Neither the doxycycline treatment nor labeling has a global influence on proteomes.

Majority of the proteins show no difference in abundance after doxycycline treatments in the replicate SILAC experiments. The H/L values represent the ratio of protein abundance in untreated/treated cells in the first experiment (left panel) and treated/untreated cells in the second experiment (right panel). The x-axis indicates individual ORFs and the y-axis indicates the log2-transformed values for normalized median ratios of heavy and light amino acid labeled proteins. A similar pattern was observed in both experiments suggesting that labeling *per se* did not cause changes in the proteome.

**Figure S2.** High reproducibility was observed between two replicate experiments.

Each dot represents one protein. The H/L values in the first SILAC experiment and the L/H values in the second experiment represent the ratios of protein abundance in untreated/treated cells. The correlation between these two sets of data was measured to be highly significant (Pearson's coefficent *r* = 0.896, p < 2.2e­16).

**Figure S3.** Heatmap depicting the fold change (FC) of mRNA and protein levels of all the up- and down-regulated proteins identified by SILAC experiments.

The genes were classified into unique functional categories (indicated by side color bars for clarity). If a gene was annotated to multiple functions, only the most significantly enriched category was considered. The category “others” contains genes that were not identified in our GO analyses. The color key along with histogram provides information on the color range and frequency of up- and down-regulated proteins.

**Figure S4.** The protein abundance measured using GFP fusion proteins was consistent with the SILAC result. Nine proteins were chosen from the post-transcriptionally down-regulated gene list identified in the SILAC experiments. Cells carrying the GFP fusion construct of these proteins were grown in medium with or without the Hsp90 inhibitor macbecin II and then the GFP protein level was measured using the fluorescence activated cell sorter (FACS). Data from these two experiments were observed to be highly correlated (Pearson's coefficient *r* = 0.835, p = 0.005).

**Figure S5.** The down-regulated proteome of low-Hsp90 cells is conserved.

(A) Analysis using orthologs between two closely related yeast species, *S. cerevisiae* and *C. glabrata*, shows that the evolutionary rate was lower in the down-regulated group than in the whole proteome. The evolutionary rate of the down group was consistently lower than the whole proteome background even when only nonessential genes were compared. (B) Pairwise comparisons between *S. cerevisiae* and the indicated species show lower evolutionary rates in the Hsp90 up-regulated proteins, compared to the whole proteome background. (C) The down-regulated proteins of human low-Hsp90 cells also exhibit low evolutionary rates. Orthologs between humans and gorillas or orangatuns were analyzed. “ *** ” indicates p < 0.001.

**Table S1**. The sample size, enrichment score, effect size and p-value of all statistical analyses in the current study.

**Table S2.** The gene list of the Hsp90-dependent proteome.

ORFs that change protein abundance but not mRNA levels in low-Hsp90 cells are shown in bold. Novel transcription factors identified by the present study are labeled in blue and novel kinases are labeled in red.

**Table S3.** Evolutionary rates of the Hsp90-dependent proteome in yeast and humans.

**Table S4**. Detailed results of gene ontology of the down- and up-regulated proteome in low-Hsp90 cells.
