# Supplementary material for: The Hsp90-Dependent Proteome Is Conserved and Enriched for Hub Proteins with High Levels of Protein–Protein Connectivity
Source: Genome Biol Evol. 2014 Oct 13;6(10):2851–65. doi: 10.1093/gbe/evu226 (PMC4224352; doi:10.1093/gbe/evu226)
Supplement: Supplementary Data [file supp_evu226_TableS1.docx]

**Table S1**. The sample size, enrichment score, effect size and p-value of all statistical analyses in the current study.

|  | ^a^Whole, ^b^Non-essential whole, ^c^SILAC-detected proteome (n= sample size) | ^a^Mis-regulated, ^b^Down-regulated, ^c^Non-essential down-regulated, ^d^Up-regulated proteome,  ^e^down-regulated helicase proteins (n= sample size) | P-value from ^a^one-sided two sample proportion, ^b^one-sided Wilcox rank-sum, ^c^Hypergeometric test | ^a^Enrichment score, ^b^Effect size |
| --- | --- | --- | --- | --- |
| The number of genes having human orthologs | ^a^3209 (n= 6542) | ^a^572 (n=904) | ^a^1.82 × 10^-10^ | ^a^1.29 |
| The number of essential genes | ^a^1182 (n= 6607) | ^b^179 (n=576) | ^a^1.74 × 10^-5^ | ^a^1.74 |
| The median for the Ka values from *S. cer* and *C. gla* comparison | ^a^0.006 (n= 4685) | ^b^0.005 (n=523) | ^b^2.21 × 10^-5^ | ^b^0.06 |
|  | ^b^0.006 (n= 3633) | ^c^0.005 (n=344) | ^b^0.07 | ^b^0.02 |
|  | ^a^0.006 (n= 4685) | ^d^0.0045 (n=280) | ^b^4.43 × 10^-6^ | ^b^0.06 |
| The median for the Ka/Ks values from *S. cer* and *K. lac* comparison | ^a^0.432 (n= 5037) | ^b^0.3876 (n=554) | ^b^1.33 × 10^-5^ | ^b^0.06 |
|  | ^b^0.4589 (n= 3928) | ^c^0.4183 (n=375) | ^b^0.01 | ^b^0.03 |
|  | ^a^0.432 (n= 5037) | ^d^0.3442 (n=299) | ^b^2.13 × 10^-7^ | ^b^0.07 |
| The median for the Ka/Ks values from human and chimpanzee comparison | ^a^0.2206 (n= 16226) | ^b^0.1512 (n=405) | ^b^3.05 × 10^-8^ | ^b^0.04 |
| The median for the Ka/Ks values from human and gorilla comparison | ^a^0.245 (n= 15404) | ^b^0.1776 (n=392) | ^b^5.74 × 10^-5^ | ^b^0.03 |
| The median for the Ka/Ks values from human and orangutan comparison | ^a^0.1888 (n= 15688) | ^b^0.1409 (n=397) | ^b^2.48 × 10^-7^ | ^b^0.04 |
| The median for the Ka/Ks values from human and macaque comparison | ^a^0.1867 (n= 15837) | ^b^0.1583 (n=403) | ^b^3.23 × 10^-5^ | ^b^0.03 |
| The median for the numbers of interaction partners | ^a^42 (n= 5811) | ^b^55 (n=575) | ^b^1.92 × 10^-9^ | ^b^0.07 |
|  | ^a^42 (n= 5811) | ^c^48 (n=396) | ^b^9.91 × 10^-5^ | ^b^0.05 |
| The median for protein half-lives | ^c^45 (n= 2784) | ^b^41 (n=406) | ^b^0.01 | ^b^0.04 |
|  | ^c^45 (n= 2784) | ^d^50 (n=206) | ^b^0.12 | ^b^0.02 |
| The median for protein molecular weights | ^c^49.4 (n= 4095) | ^b^60.1 (n=576) | ^b^1.28 × 10^-9^ | ^b^0.09 |
|  | ^c^49.4 (n= 4095) | ^d^47.8 (n=328) | ^b^0.02 | ^b^0.03 |
| The number of DNA repair genes | ^a^236 (n= 6335) | ^b^5 (n= 30) | ^*c^0.02 | ^a^4.47 |
| The number of transcription factors | ^a^196 (n= 6607) | ^#b^21 (n= 440) | ^c^0.01 | ^a^1.61 |
| *after Bonferroni correction | |  |  |  |
| # post-transcriptionally down-regulated proteome | |  |  |  |
