# Supplementary material for: The Hsp90-Dependent Proteome Is Conserved and Enriched for Hub Proteins with High Levels of Protein–Protein Connectivity
Source: Genome Biol Evol. 2014 Oct 13;6(10):2851–65. doi: 10.1093/gbe/evu226 (PMC4224352; doi:10.1093/gbe/evu226)
Supplement: Supplementary Data [file supp_evu226_TableS3.doc]

**Table S3.** Evolutionary rates of the Hsp90-dependent proteome in yeast and humans.

| ORF group | Yeast ortholog pair | Total |  |  | Non-essential |  |  |
| --- | --- | --- | --- | --- | --- | --- | --- |
|  |  | n | median Ka/Ks | p-value | n | median Ka/Ks | p-value |
| Proteome | *S. cerevisiae* vs *C. glabrata* | 4685 | 0.006 |  | 3633 | 0.006 |  |
|  | *S. cerevisiae* vs *K. lactis* | 5037 | 0.432^a^ |  | 3928 | 0.4589 ^a^ |  |
| Down | *S. cerevisiae* vs *C. glabrata* | 523 | 0.005 | 2.21 × 10^-5^ | 344 | 0.005 | 0.07 |
|  | *S. cerevisiae* vs *K. lactis* | 554 | 0.3876 ^a^ | 1.33 × 10^-5^ | 375 | 0.4183 ^a^ | 0.01 |
| Up | *S. cerevisiae* vs *C. glabrata* | 280 | 0.0045 | 4.43 × 10^-6^ |  |  |  |
|  | *S. cerevisiae* vs *K. lactis* | 299 | 0.3442 ^a^ | 2.13 × 10^-7^ |  |  |  |
| ORF group | Primate ortholog pair | n | median Ka/Ks | p-value |  |  |  |
| Proteome | *H. sapiens* vs *P. troglodytes* (Chimpanzee) | 16226 | 0.221 |  |  |  |  |
|  | *H. sapiens* vs  *G. gorilla* (Gorilla) | 15404 | 0.245 |  |  |  |  |
|  | *H. sapiens* vs *P. abelii* (Orangutan) | 15688 | 0.189 |  |  |  |  |
|  | *H. sapiens* vs  *M. mulatta* (Macaque) | 15837 | 0.187 |  |  |  |  |
| Down | *H. sapiens* vs *P. troglodytes* (Chimpanzee) | 405 | 0.151 | 3.05 × 10^-8^ |  |  |  |
|  | *H. sapiens* vs  *G. gorilla* (Gorilla) | 392 | 0.178 | 5.74 × 10^-5^ |  |  |  |
|  | *H. sapiens* vs *P. abelii* (Orangutan) | 397 | 0.141 | 2.48 × 10^-7^ |  |  |  |
|  | *H. sapiens* vs  *M. mulatta* (Macaque) | 403 | 0.158 | 3.23 × 10^-5^ |  |  |  |
| Up | *H. sapiens* vs *P. troglodytes* (Chimpanzee) | 538 | 0.103 | < 2.2× 10^-16^ |  |  |  |
|  | *H. sapiens* vs  *G. gorilla* (Gorilla) | 534 | 0.161 | 2.03× 10^-9^ |  |  |  |
|  | *H. sapiens* vs *P. abelii* (Orangutan) | 546 | 0.112 | < 2.2× 10^-16^ |  |  |  |
|  | *H. sapiens* vs  *M. mulatta* (Macaque) | 551 | 0.108 | < 2.2× 10^-16^ |  |  |  |

^a^ Non-synonymous substitution rate (Ka) instead of the nonsynonymous to synonymous substitution ratio (Ka/Ks) is used in this specie pair because the number of synonymous mutations is likely to be saturated between two distant species.
